# Goblet of fire: how *Chlamydia* ignites region-specific colitis by hijacking goblet cells

**DOI:** 10.1172/JCI204928

**Published:** 2026-04-01

**Authors:** Declan F. McCole

**Affiliations:** Division of Biomedical Sciences, School of Medicine, University of California, Riverside, California, USA.

## Abstract

Crohn’s disease can occur anywhere along the small and/or large intestines, but most commonly occurs in the terminal ileum or ascending colon. Factors governing this region-specific inflammation are poorly understood. In this issue of the *JCI*, Spencer et al. used a TNF-driven mouse model of small intestinal Crohn’s disease to identify a specific bacterial pathobiont, *Chlamydia muridarum*, as a necessary and sufficient driver of region-restricted inflammation. *C*. *muridarum* triggered increased goblet cell expression of indoleamine 2,3-dioxygenase 1 (IDO1) in the mouse proximal colon, analogous to the human ascending colon. IDO1 metabolism of tryptophan stimulated increased levels of kyneurine, and suppression of this IDO1/kyneurine axis alleviated *C*. *muridarum–*provoked inflammation in the proximal colon but not the terminal ileum. Analysis of scRNA-seq datasets from patients with Crohn’s disease with ascending colon involvement also supported increased IDO1 expression in a subpopulation of crypt surface epithelial cells. The study highlights a process by which bacterial pathobionts promote region-specific intestinal inflammation.

## The importance of regionalization along the intestine

Throughout the intestinal tract, specialized epithelial cells line the intestinal mucosa and form the essential physical barrier between luminal contents and the immune cell–rich lamina propria. There are distinct regional variations among the differentiated secretory lineages of intestinal epithelial cells (IECs). Paneth cells, for instance, are only found in the small intestine, with the highest concentration in the ileum, where they play a crucial role in maintaining host-microbe homeostasis by producing antimicrobial peptides and supporting the stem cell niche (1, 2). In contrast, the proximal colon (analogous to the human ascending colon), is notable for the absence of Paneth cells and a dramatically increased bacterial load, thus marking it as a uniquely vulnerable niche for microbe-associated disease.

Goblet cells represent another vital secretory epithelial subtype, comprising at least 20%–25% of all epithelial cells in the distal colon but 10% or less of the upper small intestine (3). Goblet cells secrete protective mucus layers in the intestine that parallel regional differences in goblet cell abundance. Mucus is thickest in the colon, where it is composed of 2 distinct layers: a dense inner layer attached to the epithelial surface and a loose outer layer that resembles the mucus layer in the small intestine (4). While bacteria are found in the outer mucus layer in the colon, they do not normally penetrate the inner layer (5).

Additional regional variations are found in immune cell populations and dietary components, including antigens, along the intestine (6). The regionalization of cell types also extends to molecular-driven physiological processes governed by the intestinal epithelium, such as nutrient transporter expression, tight junction formation and tightness of the epithelial barrier, maintenance of luminal pH, bile acid absorption, and protease activity (7). This regionalization along the gastrointestinal tract is pertinent to many pathologies that show anatomically restricted patterns of distribution, ranging from pathogenic infections to chronic inflammatory conditions such as celiac disease and ulcerative colitis.

## Pathobionts in inflammatory bowel disease

Pathobionts — microbes that are normally tolerated but become pathogenic under permissive conditions — are increasingly recognized as potential triggers of region-specific or local disease. Adherent-invasive *E*. *coli* is arguably the best-studied pathobiont associated with inflammatory bowel disease (IBD) (8, 9). *Chlamydia* species represent a compelling example of a niche-adapted bacteria with pathobiont potential. Although best known as urogenital pathogens, these gram-negative bacteria can colonize the gastrointestinal tract, and certain serovars cause proctocolitis that shares some inflammatory features with IBD (10).

A key host response to intracellular *Chlamydia* is induction of indoleamine 2,3-dioxygenase 1 (IDO1), the enzyme catalyst for the initial rate-limiting step in the degradation of the essential amino acid tryptophan along the kynurenine pathway. IDO1 can both restrict bacterial growth and promote bacterial persistence (11). IDO1 also influences epithelial differentiation and immune regulation, including activation of Tregs (12). While IDO1 is known to be upregulated in inflamed IBD mucosa and is an IFN response gene, its role in region-specific disease has not been defined.

IDO1’s conformational plasticity and highly regulated catalytic activity enable it to engage in nonenzymatic functions, including reprogramming immune cells toward a highly immunoregulatory phenotype (13, 14). Intriguingly, a study utilizing mice overexpressing IDO1 in IECs revealed that IDO1 acted nonenzymatically to activate the aryl hydrocarbon receptor, which subsequently inhibited Notch1 signaling, thereby directing epithelial differentiation toward secretory lineages rather than absorptive enterocytes (12). Conversely, IDO1 induction has also been shown to promote *Chlamydia* persistence in vitro (15). Thus, the effects of IDO1 are likely heavily dependent on additional niche-specific factors.

## Microbe exposure and genetic susceptibility promote localized colitis

In this issue of *JCI*, Spencer et al. (16) investigated how pathobionts exploit region- and niche-specific properties to drive region-specific disease in the *Tnf*^ΔARE/+^ mouse, an established, TNF-driven model of Crohn’s disease that develops inflammation in the terminal ileum, with only rare and mild inflammation reported in the colon (17). When raised in a conventional (CONV) or a specific pathogen–free barrier (SPF-B) facility, these mice developed inflammation in the terminal ileum, as expected. However, CONV-housed mice also developed severe, fully penetrant proximal colitis. This inflammation appeared early and was regionally restricted to the proximal colon at 12 weeks of age, but spread to the distal colon with age (34–42 weeks). Thus, in a genetically susceptible host, environmental factors — in this case, exposure to a more diverse population of microbes — influenced the site of inflammation and recapitulated clinical presentation of Crohn’s disease in both the terminal ileum and proximal colon.

To rule out nonmicrobial environmental effects in the CONV facility, such as caging and food supply, the authors demonstrated that colitis-free *Tnf*^ΔARE/+^ or WT mice transferred from the SPF-B facility to the CONV facility did not develop colitis if they were not cohoused with mice from the CONV facility. However, if transferred colitis-free SPF-B *Tnf*^ΔARE/+^ mice were cohoused with WT or *Tnf*^ΔARE/+^ mice from the CONV facility, they developed colitis in the proximal colon. Strikingly, no inflammation occurred in WT SPF-B mice transferred to the CONV facility and cohoused with CONV-housed WT mice. The authors then showed that the CONV microbiota, and not a specifically TNF-driven microbiota, was sufficient to trigger inflammation of the proximal colon, as WT foster dams were able to transfer colitis to SPF-B *Tnf*^ΔARE/+^ pups, as well as WT adults to SPF-B *Tnf*^ΔARE/+^ adults. Notably, this effect was not restricted to the *Tnf*^ΔARE/+^ model, as similar observations occurred in CONV-housed *Il10rb^–/–^* mice, which lack the β subunit of the antiinflammatory IL-10 cytokine receptor and develop spontaneous colitis (18).

## A microbial driver of site-specific intestinal inflammation

Shotgun metagenomics of luminal contents of the proximal colon revealed that Chlamydiota were enriched exclusively in CONV-housed *Tnf*^ΔARE/+^ mice, mapping to a single species: *Chlamydia muridarum*. SPF-B mice were *Chlamydia* negative, whereas CONV-housed mice were uniformly positive. Moreover, while *C*. *muridarum* was more abundant in CONV-housed *Tnf*^ΔARE/+^ mice than in WT mice, its relative abundance in WT mice declined with age, suggesting that WT mice could suppress *C*. *muridarum* colonization over time, but *Tnf*^ΔARE/+^ mice were unable to restrict its growth.

Microbial transfer of disease was indicated by cohousing experiments that transmitted both *C*. *muridarum* and colitis. Immunofluorescence staining of *Chlamydia* major outer membrane protein showed abundant intracellular inclusions in the proximal colon epithelium, with decreasing intensity toward the distal colon of CONV-housed *Tnf*^ΔARE/+^ mice. *C*. *muridarum* appeared to preferentially infect luminal surface absorptive enterocytes, with rare infection of secretory epithelial lineages (enteroendocrine, goblet, and tuft cells). This points to not only regional specialization, but also epithelial subtype specialization regarding initial colonization.

Additional approaches confirmed *C*. *muridarum* as a driver of inflammation. Doxycycline treatment eliminated *C*. *muridarum* and dramatically reduced inflammation in the proximal colon, but had no effect on ileitis in CONV-housed *Tnf*^ΔARE/+^ mice, providing indirect support for the region-specific influence of *C*. *muridarum* on inflammation. Direct evidence was provided by experiments showing that oral gavage of *Chlamydia*-free *Tnf*^ΔARE/+^ mice with a GFP-tagged *C*. *muridarum* strain induced persistent colonization, reduced weight gain, and provoked proximal colitis identical to that seen in CONV-housed mice. Importantly, inoculation did not alter global microbiome composition, demonstrating that *C*. *muridarum* alone was sufficient to cause inflammation in the proximal colon without causing global shifts in the gut microbiota.

## Goblet cells as facilitators of inflammation

To identify potential mechanisms involved in this niche-specific inflammatory response, the authors performed scRNA-seq analysis of proximal colon epithelia of WT and *Tnf*^ΔARE/+^ mice from SPF-B and CONV facilities. This analysis revealed minimal global transcriptional changes across IEC types but a striking, localized induction of IDO1 in goblet cells, especially surface goblet cells, as part of an enriched IFN response. Antimicrobial pathway responses were also increased in goblet cell subsets. Subsequent immunostaining confirmed that over 90% of IDO1-expressing cells were goblet cells, and expression was highest in the proximal colon.

**Figure 1:**
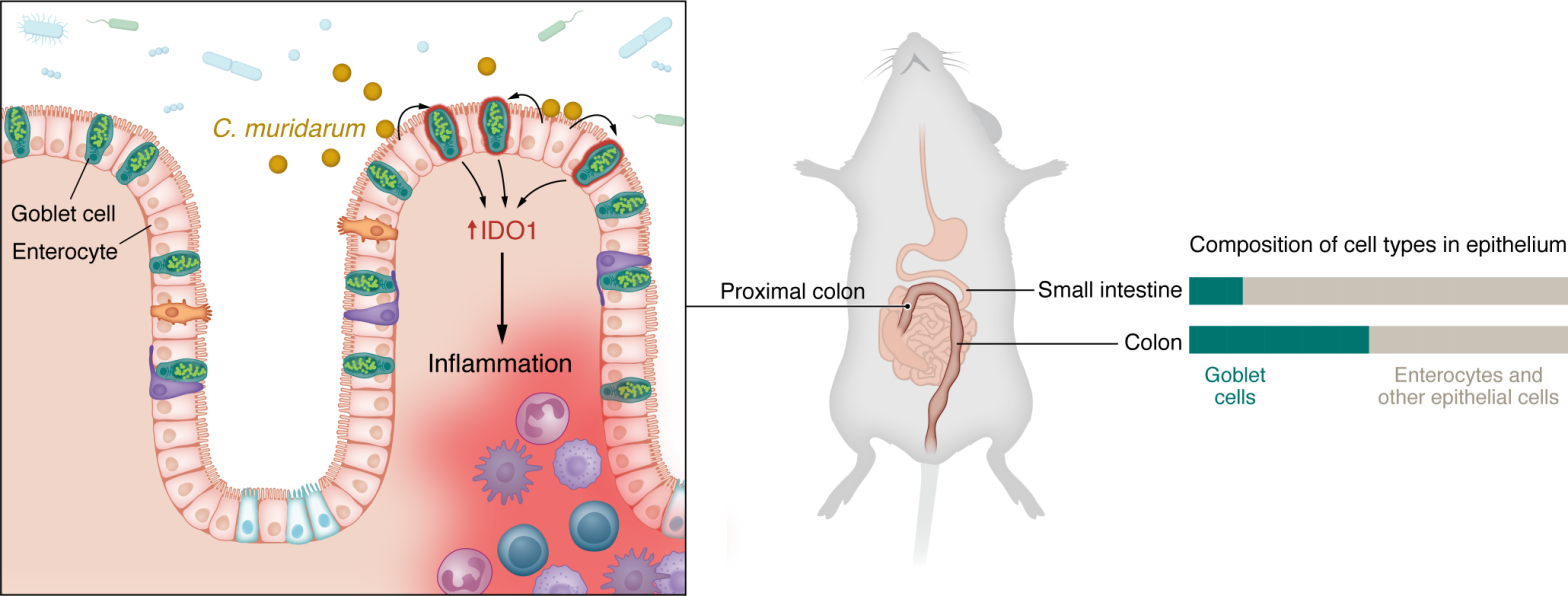
Microbial drivers of region-specific intestinal inflammation. Spencer et al. (16) reported that in a TNF-driven mouse model of Crohn’s disease, *C*. *muridarum* preferentially colonized surface absorptive enterocytes in the proximal colon (analogous to the ascending colon in humans), leading to increased production of the cytosolic, heme-containing enzyme IDO1 by neighboring goblet cells, which are abundant in this region of the gastrointestinal tract. IDO1-mediated increases in tryptophan metabolism ultimately led to increased localized inflammation in a region-specific manner.

In a series of carefully constructed experiments, the authors demonstrated reduced IDO1 expression and proximal colon inflammation despite persistence of *C*. *muridarum* colonization in an *Atoh1*-deficient mouse lacking goblet cells, mechanistically confirming that goblet cell IDO1 is an essential mediator of proximal colon inflammation. Similar findings were observed when only the crypt surface fraction of *Krt20^+^* goblet cells was depleted, thus emphasizing the critical role of this geographically restricted subset of goblet cells in the overall response to *C*. *muridarum* within proximal colonic crypts (Figure 1). Metabolomic analyses also showed elevated kynurenine/tryptophan ratios in inflamed proximal colon, thus confirming increased enzymatic activity of IDO1 in this system. The authors interrupted this mechanism by placing CONV-housed *Tnf*^ΔARE/+^ mice on a tryptophan-deficient diet, which significantly reduced proximal colon inflammation compared with *Tnf*^ΔARE/+^ mice on a control diet.

## Proximal colon inflammation occurs independently of ileitis

Next, Spencer et al. (16) used a TNF-intermediate mouse model with TNF protein expression at an intermediate level between that of WT and *Tnf*^ΔARE/+^ mice and showed that inflammation in the proximal colon occurred without ileitis. In addition, in *Tnf*^ΔARE/+^ mice, clearance of *Chlamydia* eliminated inflammation in the proximal colon but not the terminal ileum, while Paneth cell ablation had no effect on proximal colon disease or *Chlamydia* colonization. This indicated that on a background of increased TNF production, *C*. *muridarum–*driven inflammation in the proximal colon was regionally autonomous and not downstream of ileal pathology or any defect in ileal Paneth cell antimicrobial peptide production.

## Application to site-specific inflammation in patients with Crohn’s disease

Finally, the authors compared human scRNA-seq datasets and identified that IDO1 expression was restricted to ascending colon epithelial cells in patients with Crohn’s disease who had active or prior ascending colon inflammation. Immunofluorescence confirmed epithelial IDO1 protein expression in inflamed ascending colon but not in noninflamed ascending colon nor in the terminal ileum. IDO1 was almost exclusively expressed in a population of LCN2-, NOS2-, and DUOX2-expressing epithelial cells, termed LND cells, that were identified and associated with active Crohn’s disease in the ascending colon but not the terminal ileum. This served to correlate the findings of IDO1-overexpressing surface epithelial subsets localized to the ascending (proximal) colon in mouse models with human Crohn’s disease active in the ascending colon.

Altogether, Spencer et al. (16) reveal a compelling multifaceted mechanism by which a bacterial pathobiont manipulates a host cell normally associated with mucosal protection, to instead subtly modify the local environment and provoke a region-specific inflammatory response.

## Remaining gaps

Despite the study’s comprehensive mechanistic insights, several open questions remain. While the broader significance of this study is the careful elucidation of a mechanistic pathway whereby a specific pathobiont modulates host expression of a metabolic biomarker (IDO1) to drive regional events in Crohn’s disease, no directly analogous human bacterium has been confirmed to localize or induce the same effects as *C*. *muridarum* in the human ascending colon of patients with Crohn’s disease. The nature of the signal from the *Chlamydia*-colonized enterocyte to the target goblet cell, leading to IDO1 upregulation, also remains unknown. Moreover, the sequence of downstream immune cell responses arising from IDO1-mediated tryptophan depletion and how kynurenine metabolites shape localized inflammation in the ascending colon remain to be determined.

While murine colitis was shown to be goblet cell dependent, inflammation in the ascending colon of patients with Crohn’s disease was associated with increased IDO1 in LND cells, a nongoblet, proinflammatory cell type that emerges in Crohn’s disease (19). Further investigation of this cell type in human models is important for a more nuanced understanding of niche- and region-specific inflammation. Overall, this study supports a model for regional inflammation in Crohn’s disease whereby pathobionts trigger proinflammatory signaling pathways in epithelial cells in the setting of both genetic susceptibilities and permissive environmental contexts to drive chronic disease, though the specific pathobiont/cell type pairing may differ between mice and humans.

More broadly, although it is known that the proximal colon has a more penetrable mucus layer with distinct properties from more distal colonic mucus layers (20, 21), as well as a higher microbial load and a different luminal environment than the terminal ileum (22), the precise anatomical or molecular features that make this region uniquely vulnerable for *C*. *muridarum* colonization remain incompletely understood.

## Future applications

Spencer et al. (16) established an attractive model of proximal colon–restricted Crohn’s-like inflammation that addresses a major gap in preclinical research. Assuming the model is widely transferrable to other CONV versus SPF-B vivaria at different institutions, it may prove a valuable resource to further explore mechanistic and therapeutic interventions for proximal colon–specific Crohn’s disease.

The therapeutic implications for these findings argue strongly for region-specific therapeutic strategies in Crohn’s disease. This argument may be particularly germane to the pursuit of therapeutic targeting of IDO1 (or its downstream pathways) to alleviate its contribution to ascending colon–predominant Crohn’s disease without compromising its homeostatic roles that may be required to restrict inflammation in other parts of the intestinal tract. In addition, epithelial IDO1 expression, especially in LND cells, could potentially serve as a molecular biomarker for confirming ascending colon involvement and/or as a predictive marker for disease course or treatment response.

## Funding support

This work is the result of NIH funding, in whole or in part, and is subject to the NIH Public Access Policy. Through acceptance of this federal funding, the NIH has been given a right to make the work publicly available in PubMed Central.

- NIH grants 2R01-DK091281, 1R01AI153314, 1R01DK138456, and 1R01DK130373.

## Version 1 04/01/2026

Electronic publication
